# An 18-year Odyssey: navigating the complex path of prolactinoma management: a case report

**DOI:** 10.1186/s40842-025-00218-8

**Published:** 2025-04-10

**Authors:** Firdhous Alimathunisa Abdul Kather, Ariel Barkan, Shafaq Khairi

**Affiliations:** Division of Metabolism, Endocrinology and Diabetes, University of Michigan, Ann Arbor, MI USA

**Keywords:** Prolactinoma, Refractory tumor, Immunotherapy, Pembrolizumab, Case report

## Abstract

**Background:**

Prolactinomas are the most common type of pituitary adenomas, typically managed with dopamine agonists. However, some cases are refractory to standard therapies, posing significant clinical challenges. This case highlights the complexities of managing an aggressive macroprolactinoma resistant to conventional treatments and explores the use of immunotherapy as a novel intervention.

**Case presentation:**

A 54-year-old male with a history of hypertension and type 2 diabetes mellitus presented with erectile dysfunction and low libido, leading to a diagnosis of prolactinoma. Over an 18-year period, he underwent multiple interventions, including cabergoline therapy, four transsphenoidal surgeries, radiation therapy, and various pharmacotherapies. Despite these, he had refractory disease with markedly elevated prolactin levels and tumor growth. In the final stages of the disease, pembrolizumab immunotherapy was attempted. Unfortunately, the patient’s condition continued to deteriorate, ultimately leading to hospice care and death at the age of 71.

**Conclusions:**

This case underscores the challenges associated with managing refractory prolactinomas and highlights the need for innovative therapeutic strategies, including immunotherapy. Further research is essential to establish the role of emerging treatments in improving outcomes for patients with aggressive pituitary adenomas.

## Background

Prolactinomas represent the most common type of pituitary adenoma, typically managed with dopamine agonists such as cabergoline. However, some cases are refractory to standard therapies, posing significant clinical challenges. Here, we present a case of an 18-year journey of a patient with a refractory aggressive macroprolactinoma, highlighting the complexities in management and the exploration of novel therapeutic avenues.

## Case presentation

A 54-year-old male with hypertension and type 2 diabetes mellitus initially presented to his primary care provider (PCP) with erectile dysfunction and low libido. Laboratory tests revealed an elevated prolactin level, and imaging demonstrated a sellar mass, suggesting prolactinoma. Without access to his initial records, the details of early management remain unclear. He was started on cabergoline as standard medical therapy, which he took for two years with limited response. He subsequently underwent transsphenoidal surgery (TSS), where pathology unexpectedly indicated a subarachnoid cyst rather than a pituitary adenoma. With this surprising finding, his medical team discontinued cabergoline, believing there was no underlying prolactinoma. Unfortunately, no structured follow-up was arranged, and prolactin levels were not reassessed in the subsequent years. During this time, he developed primary hyperparathyroidism and a multinodular goiter, requiring near-total parathyroidectomy and total thyroidectomy. Despite these new endocrine diagnoses, his pituitary status remained unmonitored.

**Fig. 1:**
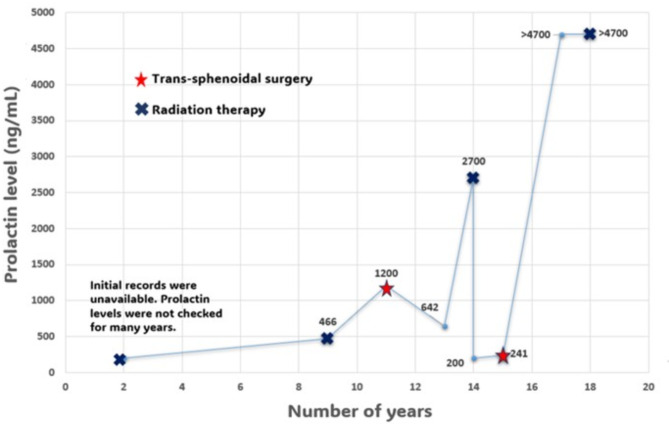
Graph showing prolactin oscillations and treatment trends

Seven years later, he presented with left-sided vision loss. MRI revealed a 5.7 cm pituitary macroadenoma and the prolactin level was 466 ng/mL (9914.89 mIU/L, Fig. 1) consistent with a prolactinoma. Cabergoline therapy was restarted, and the dose rapidly escalated over the next few months due to a lack of biochemical control. The course of treatment was complicated by a sudden cerebrospinal fluid (CSF) leak, necessitating a second TSS, which was complicated by a left temporal lobe hemorrhage and central diabetes insipidus (DI). Pathology confirmed an adenoma (Grade 1). Given his history of prolactinoma and primary hyperparathyroidism, genetic testing for multiple endocrine neoplasia type 1 (MEN-1) was performed, but no pathogenic mutation was identified. Post-operatively, his prolactin levels remained uncontrolled requiring continued therapy with cabergoline. Despite dose escalation of cabergoline over the next two years to 4.5 mg per week, prolactin levels continued to rise, reaching 1200 ng/mL (25531.91 mIU/L, Fig. 1), prompting fractionated radiation therapy.

**Fig. 2:**
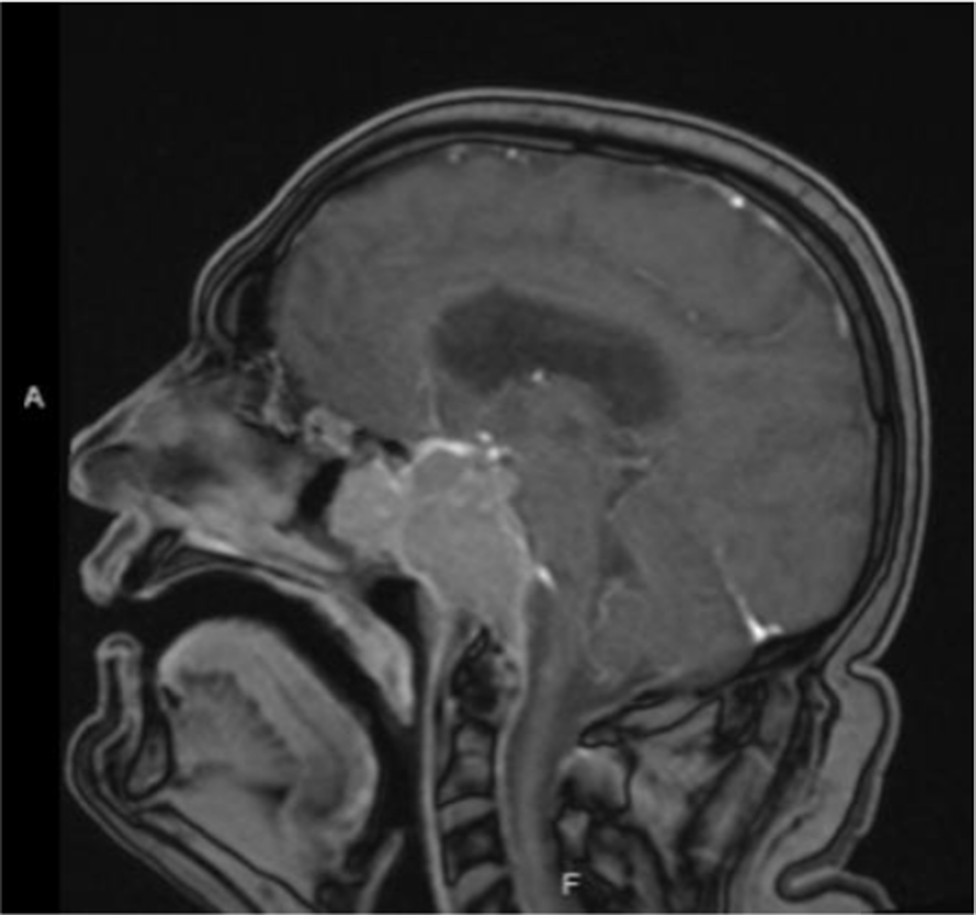
MRI pituitary

**Fig. 3:**
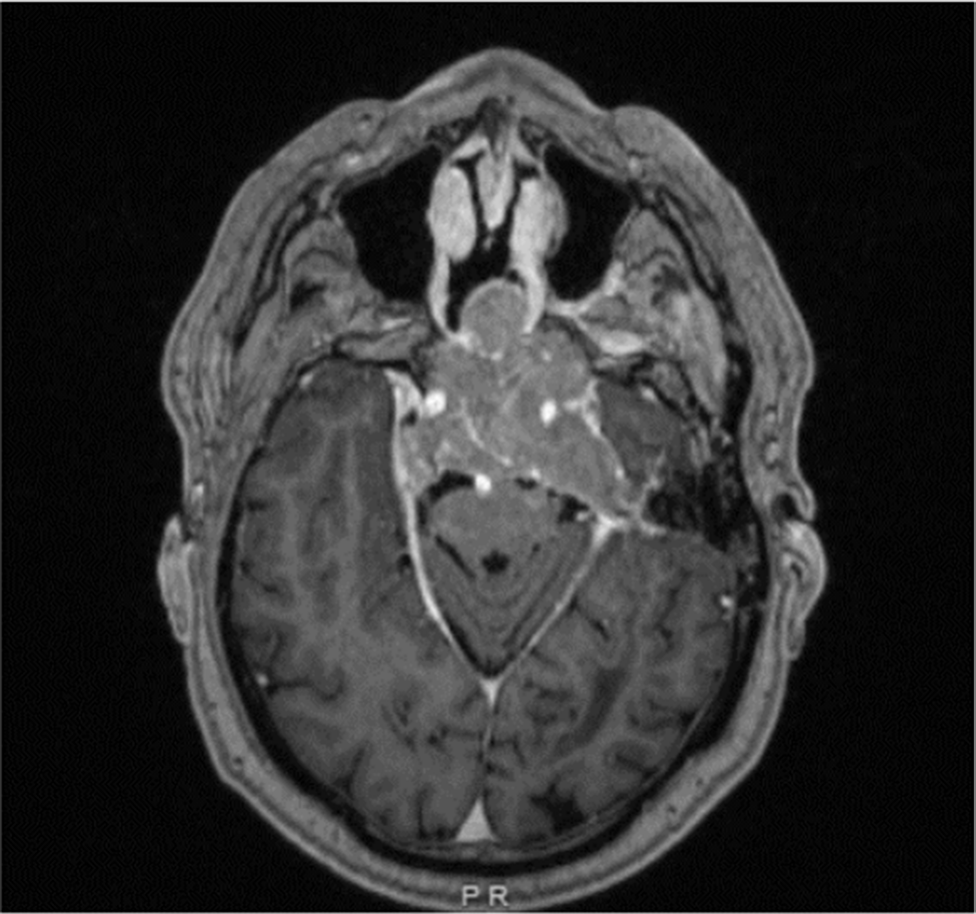
MRI 2 days preop

Post-radiation, his tumor size and prolactin levels (642 ng/mL or 13659.57 mIU/L, Fig. 1) remained stable on lower doses of cabergoline (1 mg per week). However, two years later, he presented with headaches and blurred vision. Imaging demonstrated an increase in tumor size to 2.8 × 3.9 cm, with a prolactin level of 2700 ng/mL (57446.80 mIU/L, Fig. 1). A third TSS was performed, and pathology revealed an aggressive prolactinoma with a Ki-67 index approaching 50%. Following surgery, he was maintained on cabergoline 1.5 mg twice weekly, but despite treatment, the tumor continued to grow, and prolactin levels remained elevated at 241 ng/mL (5127.66 mIU/L, Fig. 1). He underwent stereotactic radiation therapy. An echocardiography ruled out valvular heart disease associated with long-term cabergoline use. Over time, he developed worsening headaches, diplopia, and vision loss in his right eye. MRI revealed further tumor progression (5 × 3 cm) with invasion into the cavernous sinus and tracking along the trigeminal nerve (Fig. 2) and prolactin levels were > 4700 ng/mL (> 100000 mIU/L, Fig. 1). His cabergoline dose escalated to 7 mg per week. Despite being on his maximally tolerated dose of cabergoline, his symptoms did not improve, necessitating a fourth endoscopic debulking surgery (pre-op MRI: Fig. 3) with partial improvement in his vision of the right eye and third nerve palsy. Pathology confirmed an aggressive lactotroph adenoma with neuroendocrine features, a high Ki-67 index of up to 30%, and p53 positivity in 20% of cells. Post-operatively, cabergoline was continued at 1 mg daily. Despite aggressive management, his prolactin levels remained poorly controlled (> 4700 ng/mL or > 100000 mIU/L, Fig. 1), with new onset left-side vision loss.

As conventional therapies had been exhausted, immunotherapy with the checkpoint inhibitor pembrolizumab was initiated, though temozolomide with capecitabine was also considered. He received only one dose of pembrolizumab. However, due to tumor progression and intractable headaches, he opted to terminate further therapy and enrolled in hospice, passing away a week later.

## Discussion

### Introduction

Prolactinomas are the most prevalent type of pituitary tumors, accounting for a substantial proportion of functional pituitary neoplasms. Characterized by uncontrolled proliferation of lactotroph cells and subsequent prolactin hypersecretion, it significantly burdens affected individuals. With prevalence and incidence rates estimated at approximately 50 per 100,000 and 3–5 new cases per 100,000 individuals annually, its clinical relevance and impact are undeniable. Diagnosis typically hinges on elevated serum prolactin levels while other pituitary axes are within normal limits, substantiated by imaging modalities such as MRI or CT scans to ascertain tumor presence and size. Differential diagnosis is imperative to exclude other etiologies of hyperprolactinemia. Management strategies predominantly center on dopamine agonists, with cabergoline emerging as the preferred choice due to its superior efficacy and tolerability compared to bromocriptine [1, 2].

### Surgery and radiation therapy

However, despite these therapeutic advances, a subset of prolactinomas prove recalcitrant to conventional treatments, necessitating consideration of surgical and radiotherapeutic interventions. Surgery for hyperprolactinemia is typically reserved for specific scenarios where medical therapy with dopamine agonists has proven ineffective or where there are complications associated with the tumor. Some indications for surgical intervention include resistance to medical therapy, tumor compression effects, tumor growth despite treatment, intolerance/ adverse effects to medications, desire for fertility, and rarely cystic/ hemorrhagic transformation. Radiation therapy for prolactinoma is indicated when there is incomplete tumor resection, tumor recurrence, surgical inaccessibility, or aggressive and tumor behavior. It’s important to note that deciding to proceed with surgery or radiation therapy in hyperprolactinemia should be made in consultation with a multidisciplinary team, including endocrinologists, neurosurgeons, and other relevant specialists, considering individual patient factors, tumor characteristics, and treatment goals. Nonetheless, such approaches have potential complications, including new-onset hypopituitarism, cerebrospinal fluid leaks, vascular injury, and visual field compromise [1].

### Aggressive prolactinomas

Aggressive prolactinomas represent a subset of prolactin-secreting tumors distinguished by their aggressive growth pattern and resistance to conventional therapies. While comprising only 15% of prolactinomas, these tumors pose significant diagnostic and therapeutic challenges due to their radiologically invasive nature, rapid growth rate, and high recurrence potential. Notably, they exhibit limited responsiveness to dopamine agonist treatment at maximal doses, as well as surgical resection or radiation therapy. Moreover, the exceedingly rare prolactin-secreting carcinomas, constituting a mere 0.2% of adenohypophyseal tumors, underscore the rarity and complexity of aggressive transformation in prolactinomas. Diagnostic identification of aggressive prolactinomas is further compounded by the absence of specific imaging markers and reliance on histopathological assessment, necessitating the availability of tumor tissue. Pathological markers such as a Ki-67 index exceeding 3%, mitotic activity exceeding 2/10 high-power fields, and positive p53 expression indicate aggressive tumor behavior. Additionally, molecular markers including DRD2 gene variants, altered expression levels of PRDM2, Filamin A, or PRB3, and dysregulated TGF-β1/Smad3 signaling pathways offer insights into the molecular pathogenesis of aggressive prolactinomas [1, 3, 4].

### Therapeutic approach

Temozolomide (TMZ) emerges as a novel pivotal medical intervention in the treatment of aggressive and invasive pituitary tumors. This oral chemotherapeutic agent exerts its cytotoxic effects through the alkylation of guanine to O6-methylguanine, inducing DNA damage via base mismatch repair mechanisms. A recent study investigating its efficacy in a cohort of 166 patients with aggressive pituitary tumors and carcinomas, including 40 prolactinomas, reported encouraging outcomes. Most cases received TMZ as first-line therapy, with a notable 50% tumor regression rate (defined as ≥ 30% reduction in size) observed in prolactinomas. The standard TMZ regimen comprises cycles of 150–200 mg/m2 administered for 5 consecutive days every 28 days, with dose escalation from 150 mg/m2 in the initial cycle to 200 mg/m2 thereafter. Evaluating treatment success after a 3-month trial period enables the identification of TMZ responders. In cases exhibiting a favorable response, treatment continuation for at least 3 additional cycles is recommended, with potential extension based on clinical benefit and tolerability. However, hematological toxicity, notably as the most common and dose-limiting adverse effect, underscores the importance of vigilant monitoring and dose adjustments to optimize therapeutic outcomes while mitigating adverse events [1, 5, 6].

### Emerging investigational therapies for prolactinomas

Alternative treatments have been investigated or are now tested in clinical and preclinical models, but the experience is still too scant to draw definitive conclusions [1, 7]. These include:

- Immunotherapy: Pembrolizumab, Nivolumab, Ipilimumab [8–10].
- Hormonal treatments: Tamoxifen, Progesterone [1].
- mTOR/Akt inhibitors: Everolimus [11].
- Tyrosine kinase inhibitors: Lapatinib, gefitinib [12].
- Somatostatin analogs: Octreotide, Pasireotide [1, 13–16].
- Peptide receptor radionuclide therapy [1, 17].
- Cytotoxic drugs [1, 18].
- VEGF inhibitor: Bevacizumab [1, 19, 20].

Checkpoint inhibitors (CPIs) like pembrolizumab (anti-PD-1), nivolumab (anti-PD-1), and ipilimumab (anti-CTLA-4), work by blocking these proteins, thereby allowing immune cells (particularly T-cells) to recognize and attack cancer cells more effectively. Recent trials investigating the use of nivolumab and ipilimumab in patients with aggressive pituitary tumors (NCT04042753 and NCT02834013) have shown promising results in treating pituitary carcinomas (PC), particularly in hypermutated tumors. This was demonstrated in two patients with ACTH-secreting PC responding positively to pembrolizumab, while tumors without hypermutator phenotypes did not respond, raising questions about the role of immune markers and tumor genetics in CPI responsiveness. [8–10].

Selective estrogen receptor modulators (SERMs) lower prolactin (PRL) levels by blocking estrogen’s effects on lactotroph cells, which can otherwise raise PRL and promote cell growth; in resistant prolactinomas, SERMs like tamoxifen and raloxifene have been shown to reduce PRL levels by 20%. In rats, activation of nuclear progesterone receptors prevents estrogen-induced cell growth by reducing proliferation and increasing cell death, while membrane progesterone receptors trigger dopamine release, leading to decreased prolactin secretion via TGFβ1 activation and reduced cAMP levels [1].

The ErbB receptor family (EGFR, HER2, ErbB3, ErbB4) is expressed in lactotroph cells, promoting prolactin secretion, and inhibiting these receptors can lead to tumor regression. Since EGFR primarily affects the MAPK pathway and everolimus inhibits the PI3K/Akt pathway, combining lapatinib (EGFR inhibitor) with everolimus may enhance effectiveness, a combination shown to be well-tolerated in a phase 1 trial [11, 12].

In dopamine-resistant prolactinomas, the increased expression of somatostatin receptors (especially SSTR5 and SSTR1) leads to inhibited prolactin secretion by reducing cAMP levels, allowing for targeted therapies like octreotide and pasireotide, which can variably affect tumor size and PRL levels, while peptide receptor radionuclide therapy has shown potential but requires careful patient selection for effectiveness [1, 13–17].

Cytotoxic chemotherapies for prolactin-secreting carcinomas, such as carboplatin and combinations like lomustine/5-fluorouracil, aim to induce tumor cell death by damaging DNA or inhibiting critical cellular processes; however, these treatments have resulted in only modest tumor responses and often require discontinuation due to significant hematological toxicity [1, 18].

The potential effectiveness of the antiangiogenic agent bevacizumab in aggressive prolactinomas remains unstudied, but its role in inhibiting vascular endothelial growth factor (VEGF) has also shown promise in pituitary adenomas, where high VEGF expression is prevalent, as bevacizumab has demonstrated significant hormonal reduction and tumor stabilization in aggressive ACTH-secreting and nonfunctioning adenomas resistant to other therapies, suggesting it may also benefit prolactinomas as monotherapy or in combination with temozolomide [1, 19, 20].

## Conclusion

This case underscores the challenges in diagnosing and managing aggressive prolactinoma, particularly when initial records are unavailable. The patient’s almost two-decade-long journey was defined by therapeutic resistance, delay in recognizing the aggressiveness of the macroprolactinoma, necessitating escalating multimodal treatments, including repeated surgeries, high-dose dopamine agonist therapy, radiation, and ultimately systemic therapy. Despite these interventions, his disease remained refractory, leading to significant morbidity and mortality. Though aggressive macroprolactinomas are rare, it is imperative that they are diagnosed earlier on, so that systemic therapy can be considered to improve outcomes. The introduction of immunotherapy represents a novel approach to addressing resistant tumors, though its efficacy in prolactinomas remains uncertain. Further research is warranted to elucidate the role of immunotherapy and other emerging treatments in refractory pituitary adenomas.

## Data Availability

No datasets were generated or analysed during the current study.

## Abbreviations

ACTH: Adrenocorticotropic Hormone
CAM5: Cytokeratin Antibody Marker 5
CPI: Checkpoint Inhibitor
CT: Computed Tomography
CTLA-4: Cytotoxic T-Lymphocyte-Associated Protein 4
DNA: Deoxyribonucleic Acid
DRD2: Dopamine Receptor D2
EGFR: Epidermal Growth Factor Receptor
HER2: Human Epidermal Growth Factor Receptor 2
HPF: High-Power Field
INI1: Integrase Interactor 1
Ki-67: Antigen Kiel 67 (Cell Proliferation Marker)
MAPK: Mitogen-Activated Protein Kinase
MEN-1: Multiple Endocrine Neoplasia Type 1
mTOR: Mammalian Target of Rapamycin
MRI: Magnetic Resonance Imaging
PC: Pituitary Carcinoma
PD-1: Programmed Death-1
PI3K: Phosphoinositide 3-Kinase
PRB3: Proline-Rich Protein BstNI Subfamily 3
PRDM2: PR Domain Zinc Finger Protein 2
PRL: Prolactin
P53: Tumor protein 53 (Cell proliferation marker)
SERM: Selective Estrogen Receptor Modulator
SGLT-2: Sodium-Glucose Cotransporter-2
SSTR: Somatostatin Receptor
TGF-β1: Transforming Growth Factor Beta 1
TMZ: Temozolomide
VEGF: Vascular Endothelial Growth Factor
